# Advanced ultrasound methods to improve chronic kidney disease diagnosis

**DOI:** 10.1038/s44303-024-00023-5

**Published:** 2024-07-25

**Authors:** Susanne Fleig, Zuzanna Anna Magnuska, Patrick Koczera, Jannine Salewski, Sonja Djudjaj, Georg Schmitz, Fabian Kiessling

**Affiliations:** 1 Division of Nephrology and Clinical Immunology, RWTH Aachen University, Medical Faculty, Aachen, Germany; 2 Institute for Experimental Molecular Imaging, University hospital RWTH Aachen, RWTH Aachen University, Aachen, Germany; 3 Chair for Medical Engineering, Department of Electrical Engineering and Information Technology, Ruhr University Bochum, Universitaetsstr. 150, 44780 Bochum, Germany; 4 Institute of Pathology, University hospital RWTH Aachen, Aachen, Germany; 5 Comprehensive Diagnostic Center Aachen, University hospital RWTH Aachen, Aachen, Germany; 6 Fraunhofer Institute for Digital Medicine MEVIS, Am Fallturm 1, 28359 Bremen, Germany

**Keywords:** Diseases, Nephrology, Kidney diseases, Glomerular diseases, Computational biology and bioinformatics, Image processing, Health care, Ultrasonography

## Abstract

Chronic kidney disease (CKD) affects 850 million people worldwide and is associated with significant cardiovascular morbidity and mortality. Routine laboratory tests do not reflect early stages of microcirculatory changes and vascular rarefaction that characterise kidney fibrosis, the common endpoint of CKD. Imaging techniques that detect CKD in early stages could promote timely treatment with new drugs like SGLT2 inhibitors, thus, decreasing CKD progression and the cardiovascular disease burden. Ultrasound is the most used imaging modality in CKD, as it is non-invasive and radiation free. Initially, ultrasound imaging was applied to assess kidney macro-morphology and to rule out ureteral obstruction. The development of higher frequency probes allowed for more detailed imaging of kidney parenchyma, and advances in Doppler ultrasound provided insights into segmental arterial flow patterns including resistive indices as an indirect measure of microcirculatory impedance, elevated values of which correlated with progressive organ failure and fibrosis. Today, low-flow detection methods and matrix probes better resolve organ parenchyma and smaller vascular beds, and contrast-enhanced ultrasound allows perfusion measurement. Particularly, super-resolution ultrasound imaging, a technology currently being in clinical translation, can characterise the microcirculation morphologically and functionally in unrivalled detail. This is accompanied by rapid developments in radiomics and machine learning supporting ultrasound image acquisition and processing, as well as lesion detection and characterisation. This perspective article introduces emerging ultrasound methods for the diagnosis of CKD and discusses how the promising technical and analytical advancements can improve disease management after successful translation to clinical application.

## Introduction

### Chronic kidney disease is common, has a high cardiovascular mortality, and non-invasive diagnostic options for early disease stages are urgently needed

Chronic kidney disease (CKD) is a silent epidemic affecting nearly 10% of the population worldwide (an estimated 850 million people). It is more prevalent in people with diabetes, hypertension, obesity and in the elderly, and its incidence is increasing accordingly. Higher CKD stage correlates with higher cardiovascular risk and mortality^1^. Kidney function is vital; its loss can partly be replaced with dialysis several times a week, or kidney transplantation and subsequent immunosuppression. However, kidney donations are lower than the demand, and waitlisted patients spend years on dialysis with a high associated mortality, often not living long enough to receive a kidney transplant.

**Fig. 1:**
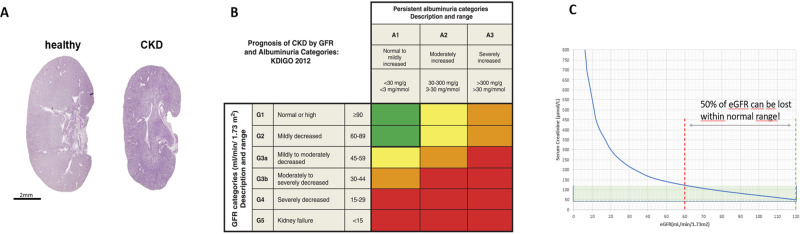
CKD grading, risk and measurement blind spot. **A** Longitudinal sections of healthy and CKD mouse kidney. **B** Prognosis of CKD by GFR and Albuminuria Categories: KDIGO 2012. **C** Hyperbolic relationship of serum creatinine and eGFR.

CKD is defined as either known kidney disease independent of kidney function (after biopsy, or inherited disease such as Alport syndrome) or impaired kidney function for more than three months independent of a known cause. CKD is graded according to the excretory function (measured by serum creatinine) and filter function (measured by albuminuria)^2^. Fibrosis, the replacement of functional tissue by scar tissue, associated with loss of small blood vessels (microvascular rarefaction)^3^, is the common pathological finding in later CKD stages, and is inversely correlated with residual kidney function (Fig. 1).

The correlation between creatinine and kidney function is not linear: a creatinine increase within the normal range can reflect a loss of >50% of kidney function. To adjust for differences in age and gender, an estimated glomerular filtration rate (eGFR) is calculated^4^ as proxy for excretory kidney function. While sensitive in low kidney function, serum creatinine is not sensitive enough to detect changes in higher or nearly normal kidney function, and definitive or early diagnosis still requires renal biopsy and histology.

An important prognostic parameter in CKD is the eGFR slope, the rate of loss of kidney function over time. Modern treatment options, such as SLGT2 inhibitors, GLP1 analogues, RAAS blockade and mineralocorticoid receptor antagonists, can slow the annual decline in kidney function and delay terminal disease requiring dialysis. Diagnosing CKD non-invasively in early stages would enable preservation of higher kidney function and thereby result in lower cardiovascular disease burden and mortality.

Acute kidney injury is a risk factor for CKD, but kidney function, measured by serum creatinine, can recover fully. This does not reflect lack of disease: In a mouse model of kidney injury using adenine diet, eGFR improved to normal after stop of adenine diet.However, the kidneys histologically showed marked fibrotic changes and no recovery^5^. Thus, early fibrotic changes can only be reliably assessed by organ biopsy and histology.

Recent advances in ultrasound technology, data processing and analysis seem to make it possible to predict organ microcirculatory function^6–12^. Hence, they could gain a high impact in the non-invasive diagnosis and better understanding of early-stage CKD.

### Non-contrast-enhanced ultrasound techniques facilitate indirect and direct assessment of renal vasculature

Loss of microcirculation is a hallmark of kidney fibrosis; however, visualisation of small blood vessels in the kidney through ultrasound has been a challenge. Colour Doppler can visualise renal segmental and interlobar arteries^13^, but not smaller vessels. Pulsed-wave-Doppler can measure blood flow velocity changes in segmental and interlobar renal arteries. Peak systolic velocity and end-diastolic velocity are needed to calculate the resistive index (RI), which increases with age as well as in kidney disease^14^. The reason is that damage in small vessels (as in small vessel vasculitis) or microvascular rarefaction (as in fibrosis) decreases the cross-sectional area and vascular compliance. While values of 0.6–0.65 are considered normal in young adults, values over 0.75 are considered pathological and have been associated with faster CKD progression^15^ and worse allograft survival in kidney transplants^16,17^. However, RI is also dependent on systemic parameters such as pulse pressure; also, high arterial stiffness and organ oedema result in increased RI. Thus, although RI is a non-invasive and reproducible parameter, it is still only an indirect measure of microvascular impedance with numerous possible confounders.

**Fig. 2:**
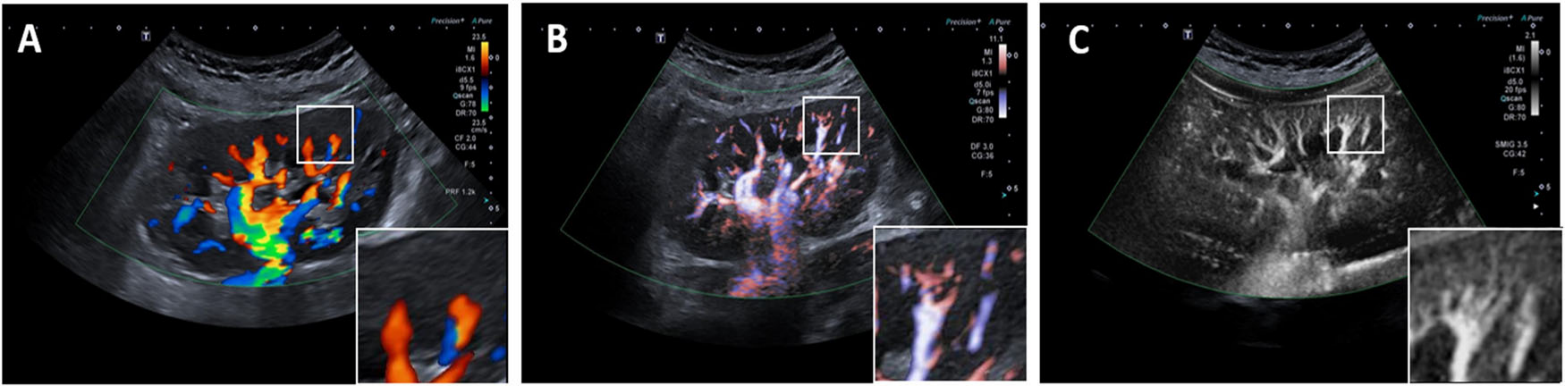
Evolution of Doppler mode in detection of smaller vascular beds. **A** Colour Doppler, (**B**) advanced dynamic flow, (**C**) SMI imaging. Insets: Cortex area and degree of measured vasculature with flow.

Recently, digital subtraction methods like B-flow imaging and low-flow Doppler (such as SMI (Superb Microvascular Imaging)^18^, microvascular imaging or “slow flow” (depending on the manufacturing company)) have shown higher sensitivity for direct detection of lower-flow and smaller vasculature in the kidney cortex (Fig. 2). For instance, in kidney tumours, SMI imaging performs almost as well as contrast-enhanced methods in identifying tumour microvasculature patterns to discern malignant lesions^19^. Furthermore, B-flow, a flow visualization method based on B-mode imaging, first patented by GE, is used in assessment of kidney transplants due to good resolution with short distance to the probe^20,21^. However, these methods can only resolve kidney structures above 300–500 µm in size, and their effectiveness depends on the used probe. Also, very low flow such as in glomeruli or capillaries cannot be resolved with these methods.

Another feature of kidney fibrosis is the deposition of extracellular matrix, resulting in increased organ stiffness. Shear Wave Elastography (SWE) or Acoustic Radiation Force Impulse (ARFI) can assess tissue elasticity, which may change with disease progression. While these methods are routinely used in liver ultrasound to grade liver fibrosis^22^, clinical utility of SWE/ARFI in kidney diseases has not yet been established. Albeit widely studied^23^, practical hurdles are the natural inhomogeneity of kidney tissue with cortex, medulla and papilla as well as large vessels and ureter structures in close proximity, allowing for only small ROIs; the organ-probe-distance is longer than in the liver context and dependent on patient BMI, and the pressure on the probe influences measurements in some elastography methods, resulting in a high inter-observer variability and low reproducibility of measurements. Possible applications include kidney tumour diagnostics. Particularly, angiomyolipomas, which are benign fat-containing tumours with softer than normal kidney tissue, thus showing differences in SWE^24^, can be identified. ARFI measurements also worked well in transplant kidneys as they are at shorter distance to the probe. In kidney transplant recipients, ARFI results correlated with the degree of fibrosis in transplant biopsies^25^. However, these protocols are difficult to integrate into standard care^25^ and the robust and reproducible measurement of mechanical tissue properties in the kidney requires further research.

In conclusion, non-contrast-enhanced ultrasound techniques facilitate the indirect and direct assessment of renal microvascular status, either by calculating RI or by imaging slow flow in small vessels. However, to improve our understanding of the mechanism of early CKD and to establish image-based biomarkers for non-invasive management of CKD progression, we need ultrasound imaging with higher sensitivity, resolution, and mechanistic expressiveness.

### Contrast-enhanced ultrasound (CEUS) may let us see more

To increase sensitivity, ultrasound contrast agents have been developed and evolved from free gas bubbles to shell-coated microbubbles in which a polymer or phospholipid shell stabilises the gas core. When exposed to cycles of high and low pressure, microbubbles undergo asymmetrical compression and expansion, which allows microbubbles to be detected and differentiated from normal tissue, which express a more linear response in contrast to the characteristic non-linear response of microbubbles^26^. The size of microbubbles (1–10 µm) allows them to pass capillaries and makes them visible in the frequency range of clinical ultrasound devices (1–15 MHz)^27^.

Intravenously injected microbubbles can be detected in vessels using the Doppler effect. Here, they either act as scatterers (like blood cells but with stronger reflection) or their fragmentation by high-intensity ultrasound pulses is registered as characteristic Doppler shift. Both increase the sensitivity of ultrasound in detecting small vessels with low flow, such as capillaries. The administration of any exogenous agent, including microbubbles for contrast-enhanced ultrasound, carries the risk of allergic reactions. Moreover, in 2008, the U.S. Food and Drug Administration (FDA) issued a safety notification about ultrasound contrast agents due to concerns about their use in patients with severe cardiovascular conditions such as myocardial infarction or pulmonary hypertension, which arose from post-market surveillance data. The clinical community debated this warning extensively. There was no conclusive evidence of more than a temporal association^28^. Recently, a new study reported an increased incidence of severe adverse events associated with the use of sulphur hexafluoride microbubbles in echocardiography. This uptrend has been examined in the context of the concurrent rise of mRNA-based COVID19 vaccines - both share polyethylene glycol as a component, which is described for its allergenic potential^29^. However, more than two decades of clinical practice and numerous safety studies demonstrated the good tolerability of microbubbles. Serious adverse events were very rare and most reported adverse events were transient and mild to moderate in severity, presenting with symptoms resembling (pseudo)allergic or physiological reactions^30–32^.

In kidney CEUS, the arterial arrival of microbubbles and the cortical enhancement and subsequent filling of the medullary pyramids can be monitored. CEUS is superior to conventional Colour Doppler ultrasound in the differentiation of renal infarction^33^ and cortical necrosis, and it can characterize kidney lesions, which are indeterminate in CT and MR. The CEUS-Bosniak classification allows assessment of cystic masses^34^ and shows a high negative predictive value with close to 100% accuracy^35^. For diagnosis of renal cell carcinoma, CEUS shows sensitivity of 100% and specificity of 86–95%^36^.

In dynamic CEUS, time-intensity curves are recorded to quantify signal enhancement over time, enabling semi-quantitative assessment of organ perfusion. After continuous infusion or during the steady-state phase, the disruption-replenishment-analysis provides quantitative values. Here, a short destructive ultrasound pulse removes the signal enhancement in the image plane and microbubble replenishment from the surrounding tissue is recorded^37,38^. Using this quantitative analysis, operator dependency is reduced, and reproducibility improved. Additionally, the destruction replenishment evaluation does not have the challenge of adequately timed image acquisition (as in DCE MRI and CT), which can be difficult in CKD patients with hyper- or hypodynamic circulation. Dynamic CEUS was shown to be capable of depicting perfusion differences in acute kidney injury^39^, after renal transplantation^40^, and in fibrotic CKD kidneys by a delayed signal enhancement and reduced peak intensity^41^. However, correlation of eGFR to dynamic CEUS parameters did not give consistent results^42,43^, and further studies are needed.

### The ups and downs of renal microvasculature imaging with super-resolution ultrasound

**Fig. 3:**
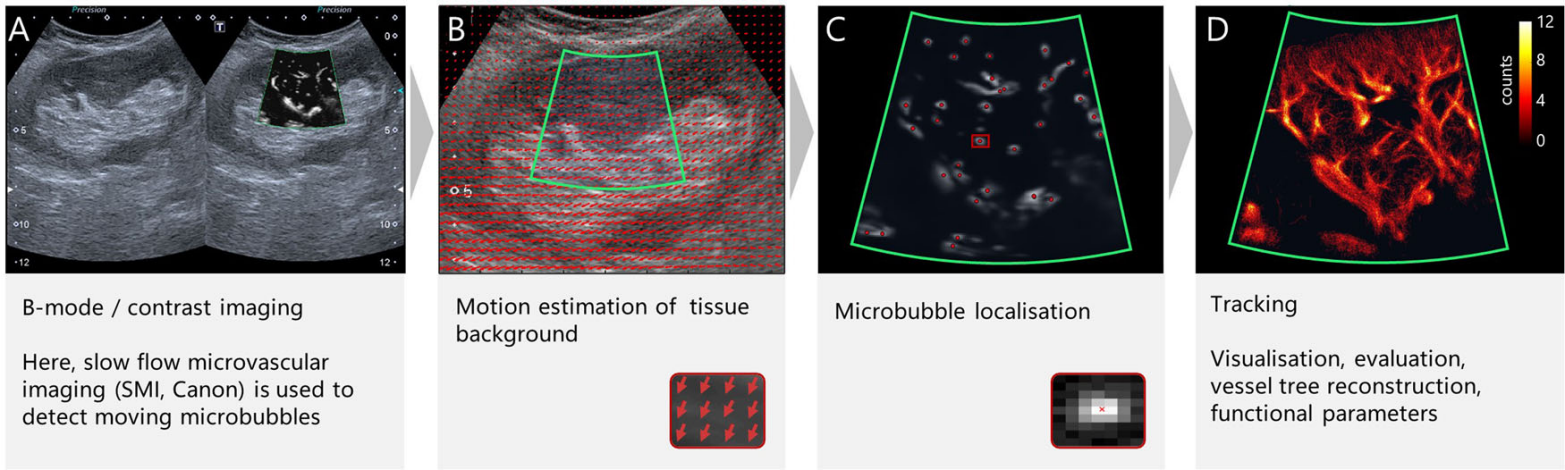
Workflow for ultrasound localisation microscopy. **A** B-mode images of a contrast bolus are acquired along with either contrast-enhanced imaging or as shown here with a slow-flow Doppler mode (SMI, Canon) to better detect microbubbles. **B** The image of tissue background is used for motion estimation and compensation. Here motion estimation was performed with the function imregdemons provided by Matlab (Mathworks, Natick, MA, USA). **C** Microbubbles are detected and localised in the contrast image. **D** Tracking of microbubbles and visualizing the tracks results in the images of super-resolved vessel. Here, an image of microbubble counts is shown. Images are from a study at RWTH Aachen University with ethical approval by the local review board.

The resolution of conventional ultrasound imaging is not sufficient to spatially resolve the microvasculature. It is limited by diffraction and has a theoretical optimal resolution of half a wavelength which is in the range of ca. 50-250 µm for frequencies between 3 and 15 MHz. The ultrasound frequency related limits were challenged by super-resolution ultrasound methods based on ultrasound localization microscopy (ULM) that were proposed independently by Siepmann et al. ^44^. and Couture et al. ^45^. in 2011. ULM is a post-processing method that is based on localizing spatially isolated microbubbles. While a microbubble is typically only 1–2 µm large, an individual microbubble is shown in the ultrasound image as a bright spot with a much larger size determined by the system’s spatial resolution. However, if microbubbles are far enough from each other so that their spots in the image do not overlap, the position of the centre of the large spot image of an isolated microbubble can be determined with high precision. The microbubble positions can either be just accumulated or tracked from frame to frame, and the microbubble velocity can be measured with algorithms as used in RADAR processing^10,44,46,7^. The tracks are then shown in high resolution images and aggregated over time revealing the microvasculature (Fig. 3).

**Fig. 4:**
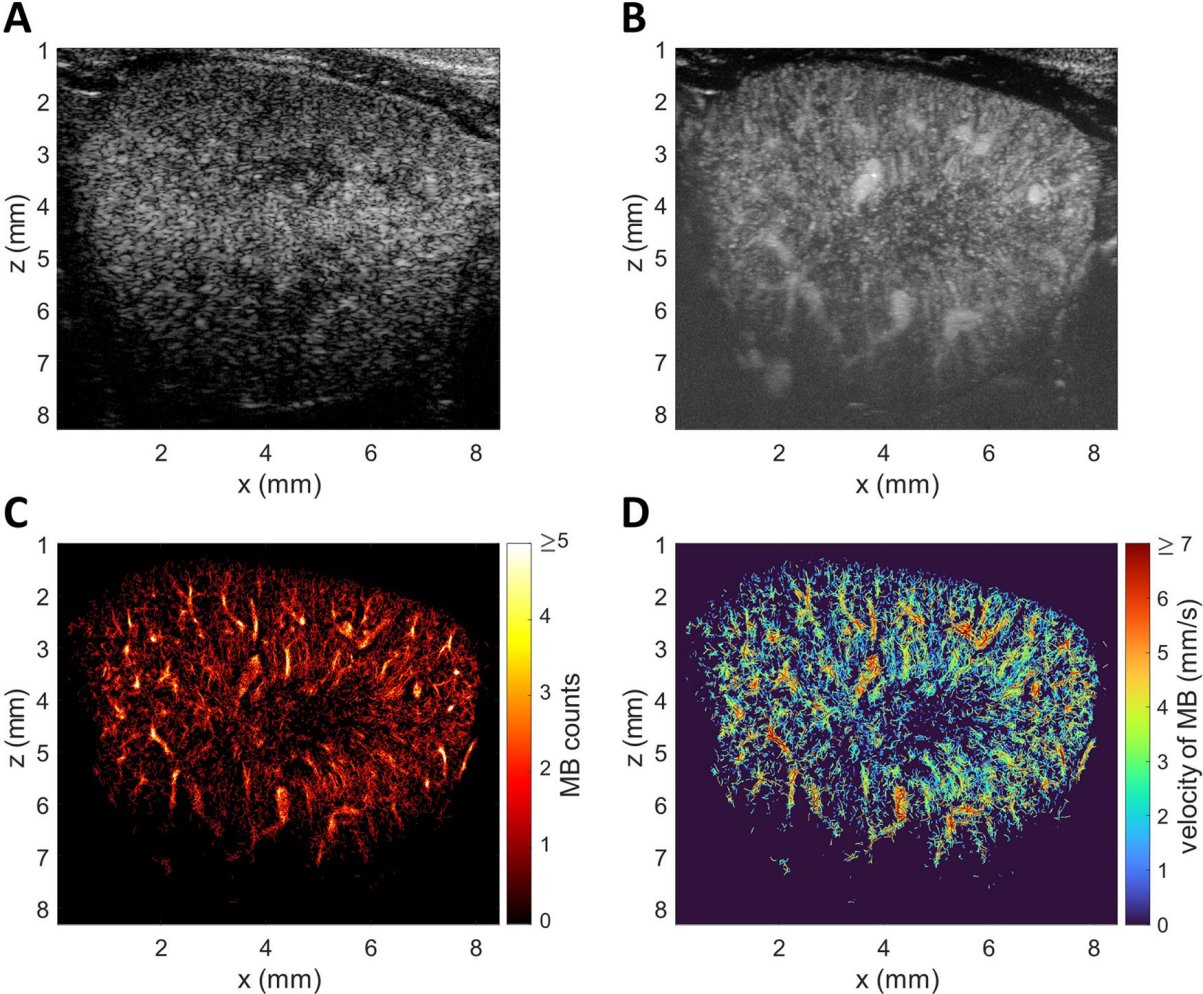
Super-resolution ultrasound microscopy images of the mouse kidney. **A** B-mode, (**B**) maximum intensity persistence image, (**C**) microbubble counts, (**D**) microbubble velocities.

ULM was applied preclinically^44^ and clinically^47^ to image the microvasculature in malignant tumours. Recent reviews^10,7^ gave an overview of further applications and the capability of ULM to show changes of the kidney microvasculature in CKD animal models (Fig. 4). ULM imaging of the kidney was first investigated by Foiret et al.^48^ in Wistar rats with a high-frame-rate research scanner (300 Hz). They demonstrated a resolution beyond the diffraction limit of 2.1 µm axially and 6.4 µm laterally, and vessels with flow velocities below 2 mm/s could be imaged. In this study, imaging was performed non-invasively and frames with out-of-plane motion were excluded while rigid in-plane motion was corrected. With a high-frame-rate research scanner (400 Hz) Qiu et al.^49^ showed a significant difference in the mean blood flow speed of interlobular arteries between hypertensive and healthy rats. The same group observed with ULM that the arterial and venous flow velocities in the renal cortex of a diabetic rat model went down compared to the control^50^. Søgaard et al.^51^ investigated changes in the density and tortuosity of kidney microvasculature in obese Zucker rats with a modified low-framerate (54 Hz) clinical scanner. They did not observe significant changes in the tortuosity, but the vessel density was reduced significantly. They pointed out that the measurement of the vessel density needs better normalization because of its dependence on acquisition time, flow rate and microbubble concentration. With the same setup Andersen et al.^12^ used ULM in kidney ischaemia/reperfusion experiments. Six blinded assessors could differentiate ULM images acquired at reperfusion after venal clamping from baseline images and 60 minutes after reperfusion no changes were seen. A further study of the same group demonstrated a decrease in the mean flow velocity under prazosin medication in the cortical arteries/arterioles and in the outer medulla descending vasa recta^52^. The use of a low-frame-rate clinical scanner in these studies led to long acquisition times of 10 minutes under constant microbubble infusion. Also, to reduce motion, the animal was opened, and the kidney was held in place with a metal retractor. Additionally, non-rigid in-plane motion correction was developed and applied^53^. It also has been observed that the detection of microbubbles in kidney images is strongly hampered by noise and different approaches for noise reduction were proposed^54,55^.

In humans, ULM of healthy kidneys was realized first with a high-frame-rate clinical scanner at 250 Hz framerate by Huang et al.^56^ and an acquisition time of only 10 s after bolus injection of Sonovue (Bracco). The study demonstrated the feasibility of ULM in patients for various organs and showed impressive detail of the kidney microvasculature^56^. However, such high framerates can currently only be achieved with few clinical scanners that are using plane-wave imaging instead of the conventional line-by-line imaging. Bodard et al.^57^ and Denis et al.^58^ investigated the microvasculature in human kidney allografts using a clinical scanner with a conventional low frame-rate between 14 Hz and 64 Hz. Both studies injected microbubbles intravenously and acquired data for 1–3 min. The authors did not implement motion compensation in their post-processing scheme. Therefore, in the studies by Denis et al.^58^ 9 of 14 and by Bodard et al.^57^ 7 of 14 patients had to be excluded from analysis due to breathing motion. This demonstrates the necessity to compensate for breathing motion or to reduce the acquisition times allowing breath holding. Bodard et al.^57^ stated that ULM identified vessels two to four times thinner than it is possible with Doppler modes. In the seven analysed patients, the mean diameter of smallest analysable vessel was reported to be 0.3 ± 0.2 mm and blood velocities were determined for the cortical region. Denis et al.^58^ additionally characterized the tracks of individual microbubbles in externalized rat kidneys and human kidney allografts. They calculated a measure for the straightness of a track being minimal for a straight linear path. Using this parameter, they identified regions with a local retention of microbubbles and hypothesized that these are locations of glomeruli. The statistical properties of their spatial distribution were analysed and compared with µCT, and results support this assumption.

While the preclinical and first clinical studies on ULM of the kidney vasculature are promising, several obstacles must be overcome for its clinical application. For instance, the degree of reconstruction of the microvessels depends on the observation time, the flow rates, and the contrast agent concentration^8,9^. In the literature, the measurement times vary from a few seconds to tenths of minutes^7,10^ resulting in a different amount of resolved microvessels^9^. Standardization of measurements is needed to derive reproducible quantitative parameters from ULM. Furthermore, for many preclinical studies the kidney was immobilized to avoid breathing motion and in clinical imaging often transplant kidneys are imaged, which also move less. To translate the results to clinical applications, it is necessary to tackle the artefacts caused by the movement that occurs due to breathing, heartbeat and transducer motion. Motion within the image plane can be estimated and corrected, and deep learning (DL) could help to better compensate for complex motion patterns. However, out-of-plane motion cannot be corrected in 2D imaging and thus frames with strong out-of-plane motion often are excluded.

Additionally, in 2D imaging in the elevational direction, perpendicular to the imaging plane, no super-resolution is achieved. This will project vessels within the image slice and makes the imaging of the dense parallel vessels in the kidney medulla and cortex difficult^48^. Moreover, it was shown by a comparison of ULM images of rat kidneys with µCT that several parallel vasa recta bundles could contribute to a single pixel in ULM and thus cannot be resolved^60^. In the future, this could be overcome by transducers also electronically focused in the elevational direction like 2D transducer arrays for 3D imaging or full matrix arrays for volume imaging.

The larger inter-frame time makes tracking of microbubbles more difficult and tracking methods based on motion models as proposed by Ackermann and Schmitz^46^ and Tang et al.^61^ must be applied. However, in the case of ultrasound kidney imaging, the large variation of velocities makes the application of motion models more difficult. It could be handled by a hierarchical Kalman tracker as proposed by Taghavi et al.^59^, which separates the tracking for different velocity ranges. In the future this may be solved by the availability of high frame-rate clinical scanners as well as advanced tracking using DL-based methods.

In summary, super-resolution ultrasound imaging of the kidney is evolving and holds a diagnostic potential to study microvascular changes in early stages of fibrosis non-invasively, improving our understanding of disease mechanism and allowing for early diagnosis and timely initiation of progression-delaying therapy. Also, by using the characteristic flow patterns of microbubbles passing through glomeruli, ULM could potentially identify and count nephrons non-invasively (related publications are summarized in Table 1), allowing for non-invasive estimation of perfused glomeruli in a defined cortex volume and extrapolation of kidney function. The perspective of introducing ULM in real-life clinical condition lays in a combination of technological advances of clinical ultrasound systems and artificial intelligence techniques. DL will be particularly useful for advanced motion compensation, tracking of the microbubbles and the extraction of diagnostic parameters.

**Table 1:** Studies of ultrasound localization microscopy of the kidney and their measurement conditions. Human studies are marked in green.

Paper first author	Year	Subject(s)	Frame-rate in Hz	US-System	Transducer	Contrast agent	Motion correction	Remarks
Huang^56^	2021	human	250	Mindray Resona 7	Mindray L9-3U	SonoVue bolus, acquisition time < 10 s	Rigid	
Bodard^57^	2023	human	14–64	Canon Aplio i800	Canon i8cX1	SonoVue bolus injection, 1-3 min acquisition	none	Allograft kidneys
Denis^57^	2023							
		rat	400	Vantage 256	Verasonics L22-14	Sonovue Infusion/acquisition for 3 min	none	Kidney externalized and fixed with pins on plastic plate
Qiu^49^	2022	rat	400	Vantage 256	Vermon L15 Xtech	Sonovue, bolus injection, acquisition after 20 s for 15 s	Rigid	Non-invasive imaging
Zhang^50^	2023							
Taghavi^53^	2021	rat	54	BK 5000 modified clinical system	BK X18L5s	SonoVue 10 min infusion	Non-rigid, respiratory gating,	surgically exposed kidney with fixation by metal retractor
Taghavi^59^	2022							
Søgaard^51^	2022							
Andersen^12^	2020							
Andersen^60^	2021							
Andersen^52^	2022							
Foiret^48^	2017	Rat	300	Vantage 256	Philips CL15-7	Self-produced lipid microbubbles	Rigid, Respiratory gating	
Chen^61^	2020	mouse	250	Vantage 128	Verasonics L22-14	Definity bolus injection acquisition after 10 s for 4 s	Rigid	No tracking, only localization
Lei^54^	2022	macaque	500	Vantage 256	Jiarui Electronics Company L9-4	Sonovue bolus injection, 3 s acquisition time	2-stage affine and non-rigid	
Song^55^	2018	rabbit	500	Vantage	Verasonics L11-3v	Optison, bolus injection acquisition time 2 s	Rigid	

### Artificial intelligence can enrich the conventional and advanced ultrasound imaging of renal microvasculature

Recent developments in AI have significantly expanded the scope and scale of medical image analysis^62,63^. This offers the prospect of a solid and objective basis for standardizing kidney ultrasound imaging and its interpretation, with a direct impact on improving the management of CKD. Certainly, the risk of developing CKD or monitoring its progression can be assessed by building classification models based on clinical values alone^64^. However, using these features alone in a predictive model may lead to inconclusive results. This is because the outcomes of common tests, i.e. blood serum creatinine or urine protein, do not directly correlate with kidney function.

**Fig. 5:**
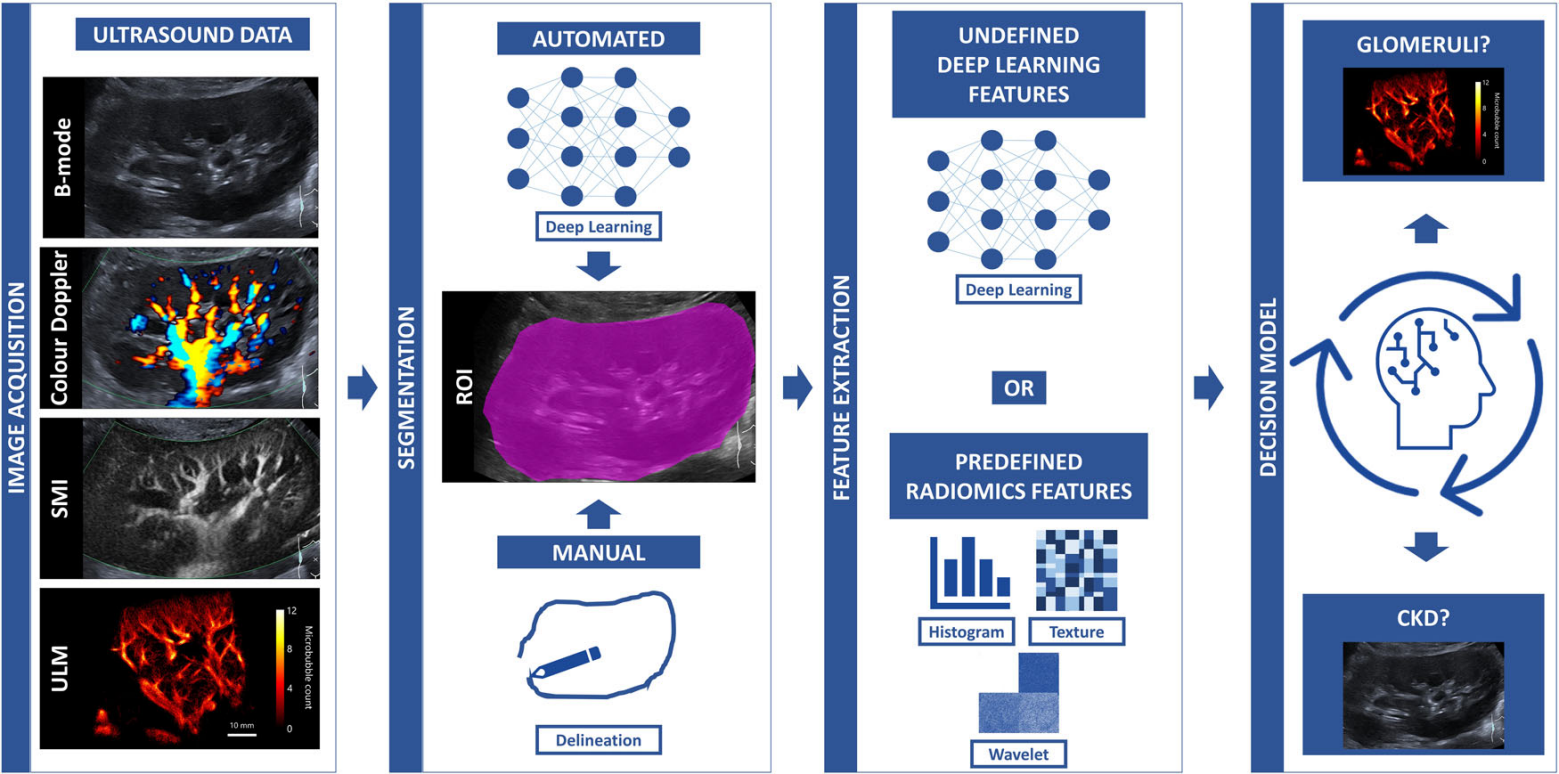
AI-based decision modelling workflow. Ultrasound data acquired by established (B-mode, colour Doppler, SMI) and emerging (ULM) ultrasound methods can be used for building AI-based decision models. First a region of interest (ROI) is selected. This is usually done by segmenting the image either automatically with e.g., deep learning or manually by delineating the ROI. Then, features are extracted from the selected ROI. Here, the features can be calculated automatically (deep learning) or by using predefined radiomics features (histogram, texture and wavelet). In the final step, the most descriptive features are selected and used to build a decision model that facilitates the diagnosis of a disease from the image.

AI-based analysis of kidney ultrasound images can facilitate the discovery of non-invasive image-based biomarkers for the assessment of kidney function (Fig. 5). Kidney ultrasound imaging is a preferred method for predicting and monitoring CKD due to its safety, convenience and affordability^65^. However, this non-invasive assessment of kidney function is not without its pitfalls. Ultrasound imaging is highly operator-dependent, making conventionally derived parameters such as kidney length, parenchymal thickness or echogenicity subjective to the reader^6^. Therefore, it would be optimal to use other strategies to interpret kidney ultrasound images, and radiomics analysis is one of them.

Radiomics is a method that supports the conversion of image data into quantifiable features^66^. To calculate radiomics features, the region of interest must first be selected, and in the case of CKD monitoring, this is kidney parenchyma. Features are then obtained using first order (histogram) and second order (texture) statistics, but also wavelet transformation to obtain the relationships between pixels hidden to the human eye. For example, wavelet features are sensitive to the directionality of speckle pattern, though they can be useful in classifying fibrosis in CKD, as its presence in the kidney parenchyma can alter the speckle pattern in the b-mode ultrasound image^67^. Additionally, descriptive image-based features can be derived from filtered or fractal ultrasound images to achieve location-sensitive classification of CKD^68,69^.

Radiomics is certainly a powerful tool which could facilitate the ultrasound-based analysis of kidney function for CKD prediction and management. For instance, a preliminary clinical study demonstrated that ultrasound-based radiomics features allowed biopsy-free differentiation between membranous and IgA nephropathy^70^. Another study showed that combining radiomics with common clinical CKD biomarkers improves the predictive performance of a CKD risk assessment classification model^71^. Nevertheless, the limitation of radiomics lays in the predefined nature of the extracted features.

DL alleviates the bottlenecks of conventional radiomics analysis. It can be employed to guide medical image processing from segmentation to disease prediction without the need for user interaction. Recent studies showed that DL-based automatic segmentation of kidney in B-mode ultrasound images can be performed with high accuracy (94% similarity to radiologist’s delineations)^72^. In addition, DL-based assessment of kidney function can discriminate between healthy and CKD patients, as well as between stages of CKD itself, and it can also be used to estimate AI-based glomerular filtration rate from B-mode images^62,73^. However, DL-based image analysis is a data-driven approach and collecting and storing medical data is still challenging. Therefore, the prospect of standardizing DL for CKD management lies in using data augmentation, transfer learning, or federated learning^62,64^. Additionally, published studies relay mostly on static B-mode images, and using Doppler or contrast-enhanced data would already expand the scope of DL-based analysis^62,72,73^. Furthermore, combination of DL and super-resolution ultrasound imaging holds great promise for non-invasive visualization and assessment of micro-pathological changes in kidney tissue. Union of these two methods could result in providing information on glomerular number, perfusion and structural integrity of kidneys.

## Conclusion

Ultrasound is already the most widely used non-invasive imaging modality for the assessment of CKD. It provides a quick and inexpensive assessment of macro-morphology and some relevant functional estimates from which the eGFR can be roughly estimated. However, as ultrasound is more user-dependent compared to other imaging modalities such as CT and MRI, and thus has low reproducibility, there is a strong demand for computer-based solutions that help the physician to re-identify a tissue slice and perform precise lesion detection and size measurements for clinical routine more reliably. These tools can be supported by AI-based algorithms and new hardware such as 3D matrix probes and help to compensate for tissue motion, which is currently the biggest challenge in translating new high-resolution ultrasound imaging techniques of the kidney into the clinic. The motion caused by the doctor’s hand and patient’s breathing, heartbeat and bowel movements, is quite complex, as it occurs in multiple directions and sometimes out of plane. If this is properly addressed, even glomeruli can become visible, as shown in a study using contrast-enhanced ultrasound localisation microscopy^58^.

From a clinical point of view, however, robustness, routine suitability and costs are essential for the acceptance of a method by physicians. Although ultrasound contrast agents are well tolerated by patients, their administration complicates examinations and increases costs. Whether super-resolution ultrasound imaging will be able to work without microbubbles in the future^59^, and whether it will add value to a comprehensive AI-based analysis of morphological and Doppler imaging, remains to be investigated. Nevertheless, it is likely that ultrasound will remain the method of choice for routine imaging in CKD and has enormous potential for further development.
